# Why mindfulness matters in musculoskeletal therapies: a narrative review

**DOI:** 10.3389/fpsyg.2025.1518141

**Published:** 2025-10-17

**Authors:** Peter Bablis, Ryan R. Day, Sophia Bablis, Henry Pollard

**Affiliations:** ^1^University Research Institute of Maternal and Child Health & Precision Medicine, Athens, Greece; ^2^Universal Health, Double Bay, NSW, Australia; ^3^Faculty of Health Sciences, Durban University of Technology, Durban, South Africa

**Keywords:** mindfulness-based interventions, musculoskeletal therapy, chronic stress management, biopsychosocial model of healthcare, neuro-emotional technique, psychoneuroimmunology, mind–body interventions, stress physiology

## Abstract

This narrative review explores the integration of mindfulness-based interventions (MBIs) into musculoskeletal (MSK) care, based on the premise that addressing the mind–body interface can enhance patient outcomes. First, we outline how chronic stress affects twelve physiological systems and contributes to the onset and persistence of MSK conditions. Second, we synthesise evidence showing that MBIs mitigate these stress effects through mechanisms such as improved attention regulation, interoceptive awareness, and reduced catastrophising. Third, we highlight Neuro-Emotional Technique (NET) as a practitioner-facilitated hybrid-MBI exemplar that targets unresolved emotional stress patterns and aligns with the biopsychosocial model of healthcare. We also note contraindications via red and yellow flag considerations, emphasising the importance of careful patient selection and responsible application. Taken together, this review provides a rationale for incorporating MBIs as adjuncts to conventional therapies, supporting recovery, fostering resilience, and advancing patient-centred MSK care.

## Introduction

Healthcare has long grappled with the separation of mind and body, a concept deeply rooted in Western medicine’s historical and philosophical traditions. Although this separation facilitated the development of specialised treatments, it also led to a fragmented approach to patient care, overlooking the interconnected nature of mental and physical health (World Health Organization, 2024). In this narrative review, we propose that musculoskeletal (MSK) practitioners consider mindfulness-based interventions (MBIs) as part of an integrative approach to care – a perspective presented to, but not widely adopted by, this audience in recent years.

The literature on the practical application of mindfulness interventions in MSK therapies remains sparse. This review therefore draws together evidence on the interdependence between psychological and physical well-being, the effects of chronic stress on twelve major physiological systems, and how MBIs may counteract these detrimental stress effects (Robinson, 2023; Canadian Mental Health Association Ontario, 2014). In view of the influence of stress on conditions commonly managed in MSK practice, considering mind–body factors forms part of a comprehensive duty of care to provide safe and effective treatment.

### Aim and scope

The aim of this narrative review is threefold. First, we outline how chronic stress contributes to MSK conditions across biological, psychological, and social domains. Second, we synthesise evidence demonstrating how MBIs mitigate stress-related mechanisms and provide a rationale for their application alongside MSK therapies. Third, we highlight Neuro-Emotional Technique (NET) as an exemplar of a practitioner-facilitated hybrid-MBI, illustrating how such approaches may be operationalised in MSK practice. Together, these aims address the impact of MBIs on health, review an example of good practice, and clarify clinical decision-making considerations for MSK practitioners.

## Background: the historical context of mind–body dualism

The dichotomy between mind and body has been a subject of philosophical debate for centuries. In the 4th century BC, Plato lamented, “the great error of our day in the treatment of the human body [is] that physicians separate the mind from the body (Maté, 2024).” Despite this, the dualistic approach became entrenched in Western medicine, heavily influenced by René Descartes’ 17th-century philosophy, which posited that the mind and body are fundamentally distinct entities. This view shaped medical practice for centuries, emphasizing the treatment of physical symptoms while often neglecting psychological aspects of health (Mantri, 2008).

However, integrative healthcare approaches, such as those found in traditional Chinese medicine, Ayurveda, and other non-Western traditions, have long recognized the inseparability of mind and body in achieving optimal health (Fan, 2017). These, and other ancient traditions and philosophies, emphasize that physical symptoms often reflect underlying psychological distress and vice versa, ultimately founding what now know as “meditation” and “mindfulness.” While agreed definitions remain elusive due to contrasting cultural foundations and ideas (Chiesa, 2013; Awasthi, 2013; Baer, 2019; Grossman, 2011), Western pioneers like Hippocrates similarly advocated for treating patients holistically, considering both physical and psychological dimensions (Mantri, 2008).

Today, specialization in medical fields often leads to a narrow focus on specific conditions, neglecting the broader context of a patient’s overall health (Mantri, 2008). Although the concept of mind–body integration is becoming more widely accepted in holistic healthcare settings, its practical application remains limited in many mainstream complementary health practices. The interdisciplinary scientific field of psychoneuroimmunology (the study of the interactions between psychological states, the nervous system and the immune system) shows that poor mental or emotional health as a result of chronic or acute stress frequently overlaps with musculoskeletal pain and illness (Australian Institute of Health and Welfare, 2024; Mental Health Foundation UK, 2024b; Barbe and Barr, 2006; Aviña-Zubieta et al., 1997; Navarro-Ledesma et al., 2024; Malterud, 2010; Ahrens et al., 2012), with each influencing the other’s severity and progression (Doan et al., 2023). In this paper, the scientific evidence is discussed in detail for the purpose of aiding recognition of the many common ways stress-related illness presents to MSK practitioners, and the role of MBIs as adjunct lifestyle and treatment interventions for MSK therapies.

### The need for a mind–body approach in MSK care

Musculoskeletal (MSK) practitioners, who focus on treating conditions related to the muscles, bones, and joints, often encounter patients with symptoms influenced by psychological factors. Conditions such as chronic back pain, fibromyalgia, tension headaches, and arthritis are frequently exacerbated by stress, anxiety, sleep loss, and emotional distress (Mills et al., 2019; Anxiety and Depression Association of America, 2024). This interconnection underscores the importance of adopting a holistic, integrative approach that addresses both physical and psychological health components (Dunn et al., 2024).

The holistic approach aligns with the biopsychosocial (BPS) model proposed by George Engel in 1977, which encourages healthcare providers to consider biological, psychological, and social factors in understanding health and disease (Engel, 1977). For MSK practitioners, this model offers a framework for addressing not just the physical symptoms but also the psychological and emotional contributors to a patient’s condition such as patient beliefs and expectations, and the influence of the interpersonal dynamics in clinical encounters on health outcomes. Ultimately, this approach can promote a more comprehensive understanding of their patients’ health, fostering recovery from physical ailments and enhancing overall well-being (Borrell-Carrió et al., 2004).

Understanding how chronic stress operates across these domains provides the foundation for examining its systemic physiological effects, outlined in the next section.

### The effects of chronic stress on physical health

Stress is defined as a state of threatened homeostasis following exposure to adverse forces (Chrousos and Gold, 1992). Acute stress (lasting minutes or hours) may be adaptive and even beneficial (Dorn and Chrousos, 1993; Rudland et al., 2020). Chronic stress, however, persists for days, weeks, or months (Olff, 1999) and is a significant factor in the onset, progression, and exacerbation of many health conditions (American Psychological Association, 2022; Mental Health Foundation UK, 2024a; American Psychological Association, 2022). When physiological demands are exceeded (Koolhaas et al., 2011), chronic stress contributes to allostatic load (Salleh, 2008; McEwen, 1998; McEwen and Stellar, 1993; Juster et al., 2010), depletes the body’s adaptive capacity (Kazakou et al., 2023), resulting in maladaptation (Stratakis and Chrousos, 1995) and poorer health outcomes (Guidi et al., 2021; Parker et al., 2022).

These effects are mediated through the Hypothalamic–Pituitary–Adrenal (HPA) axis, the body’s central stress response system, and the Psycho-Immune-Neuroendocrine (PINE) network, which integrates neural, immune, and endocrine functions to maintain health (Kazakou et al., 2023; Hardy and Pollard, 2006; Chrousos, 2009; Mariotti, 2015; Morey et al., 2015; Charmandari et al., 2005; Chrousos and Kino, 2007; González-Díaz et al., 2017). Dysregulation of these psychophysiological systems contributes to widespread health impacts including chronic illness, musculoskeletal disorders, acute and chronic pain, and organ system dysfunction that can result due to unique genetic, epigenetic and environmental factors, and the influences of the past stressful experiences of the individual (Chapman et al., 2008).

**Figure 1:**
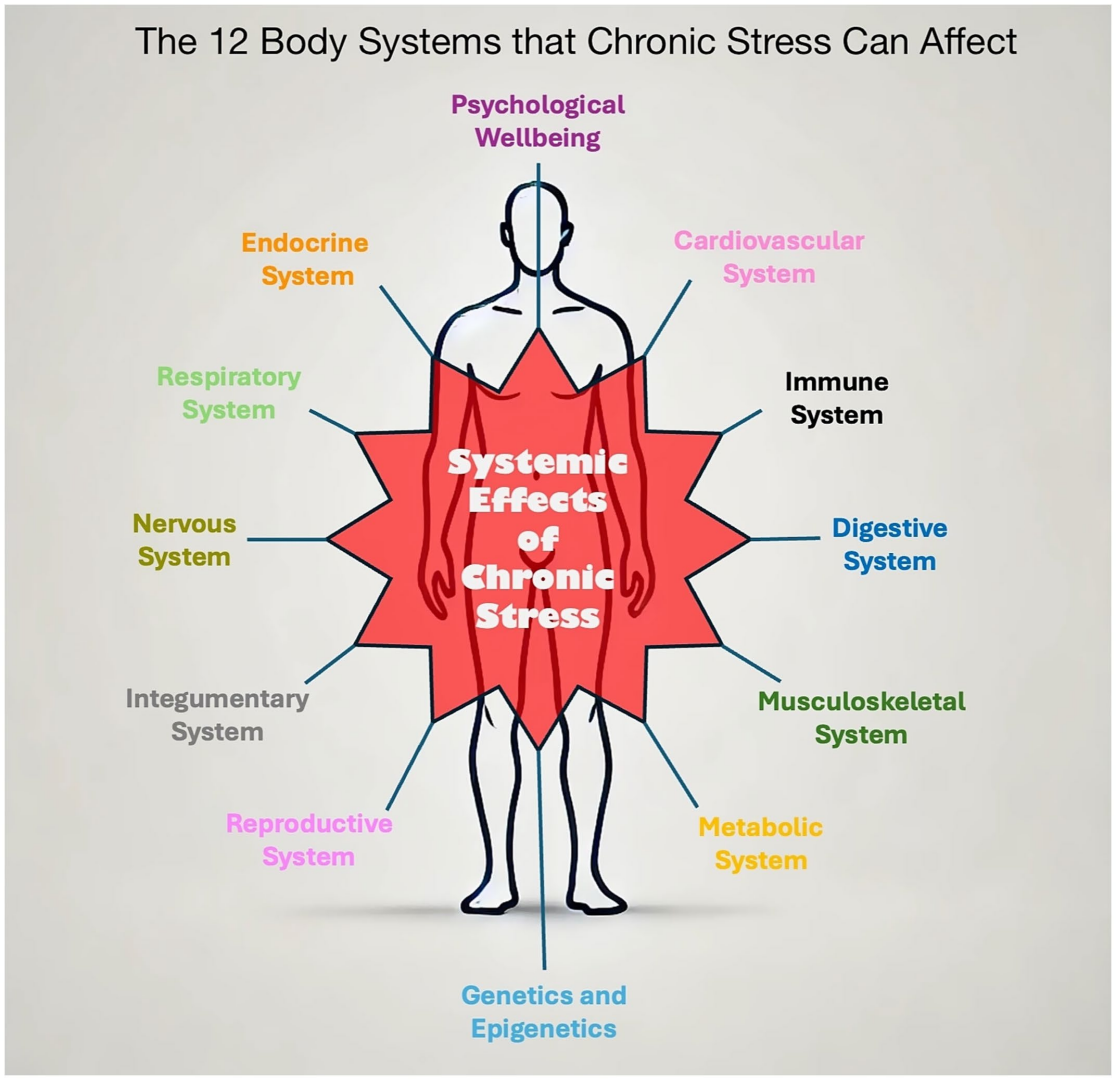
The 12 body systems that chronic stress can affect.

This is particularly relevant for MSK practitioners as chronic stress contributes to the onset and persistence of many common musculoskeletal presentations (Salvagioni et al., 2017; NIB, 2021; Schneiderman et al., 2005), including tension-type headache (Bendtsen and Fernández-de-la-Peñas, 2011), neck, shoulder, and upper-limb pain (Cagnie et al., 2007; van den Heuvel et al., 2005; Bongers et al., 2006), temporomandibular joint pain (Fillingim et al., 2013; Manfredini et al., 2003; Manfredini et al., 2004), fibromyalgia (Paschali et al., 2021; McBeth and Silman, 2001), back pain (Linton, 2000), and myofascial trigger points (Bosque et al., 2023). Chronic stress can diminish function and contribute to illness across all major body systems (Figure 1).

These systemic mechanisms provide the biological and clinical context for understanding how stress is embedded within a biopsychosocial (BPS) model of MSK care.

### The BPS model of stress in MSK care

Within the BPS model of MSK care, chronic stress functions at three levels: biologically through HPA and PINE dysregulation, psychologically through appraisal, attention, and avoidance patterns, and socially through contextual pressures such as workload or relationships. Mindfulness practices directly target these mechanisms (Maloney et al., 2024). By enhancing interoceptive awareness and attention regulation, and cultivating non-reactivity and acceptance, they can reduce muscle tension, pain sensitivity, catastrophizing, and fear-avoidance while improving adherence to active care (Todd and Aspell, 2022; Aboushaar and Serrano, 2024). This integration clarifies why stress-responsive MSK pain often improves when cognitive, affective, and behavioral dimensions are addressed alongside physical treatment (Dunn et al., 2024).

Because chronic stress operates across biological, psychological, and social domains, interventions that target multiple dimensions simultaneously are especially relevant. Mindfulness-based interventions (MBIs) represent one such approach. The following section therefore defines mindfulness and its principal categories before considering how different forms of MBIs apply to MSK practice.

### Mindfulness and mindfulness based interventions (MBIs)

American professor Jon Kabat-Zinn, creator of Mindfulness-Based Stress Reduction (MBSR) program (Kabat-Zinn, 1990) and credited by many as the founder of the contemporary mindfulness movement, defines mindfulness as, “awareness that arises through paying attention, on purpose, in the present moment, non-judgementally,… in the service of self-understanding and wisdom (Mindful Staff, 2017).” The 9 attitudinal principles of mindfulness, according to Kabat-Zinn, are: nonjudging, patience, a beginner’s mind, trust, non-striving, acceptance, letting go, gratitude and generosity (Kabat-Zinn, 2024). These attitudes and principles can be applied across a broad spectrum of practices (Maloney et al., 2024) in two broad categories: Formal (high intensity) and Informal (low intensity).

- Formal (high intensity) practices can be Dynamic (movement-based), Static (attention-based) or Hybrid practices that combine elements of both (Lutz et al., 2008; Dunn et al., 1999; Rao, 2011; Kabat-Zinn, 2003; Prakhinkit et al., 2014). They are usually facilitated by a practitioner or facilitator and often follow a given structure/process.
- Informal (low intensity) practices tend to be less structured, less time-consuming [showing benefits in as little as a few minutes (Hanley et al., 2015; Palmer et al., 2023; Strohmaier et al., 2021; Pratt, 2024)] and more easily accessible (Vilhauer, 2024), helping increase stress resilience when performed daily (Manigault et al., 2021). Informal practices have been shown to be effective in reducing stress (Sparacio et al., 2024), and can be categorised broadly as either Daily Life Awareness practices or Brief Self-Directed practices.

**Figure 2:**
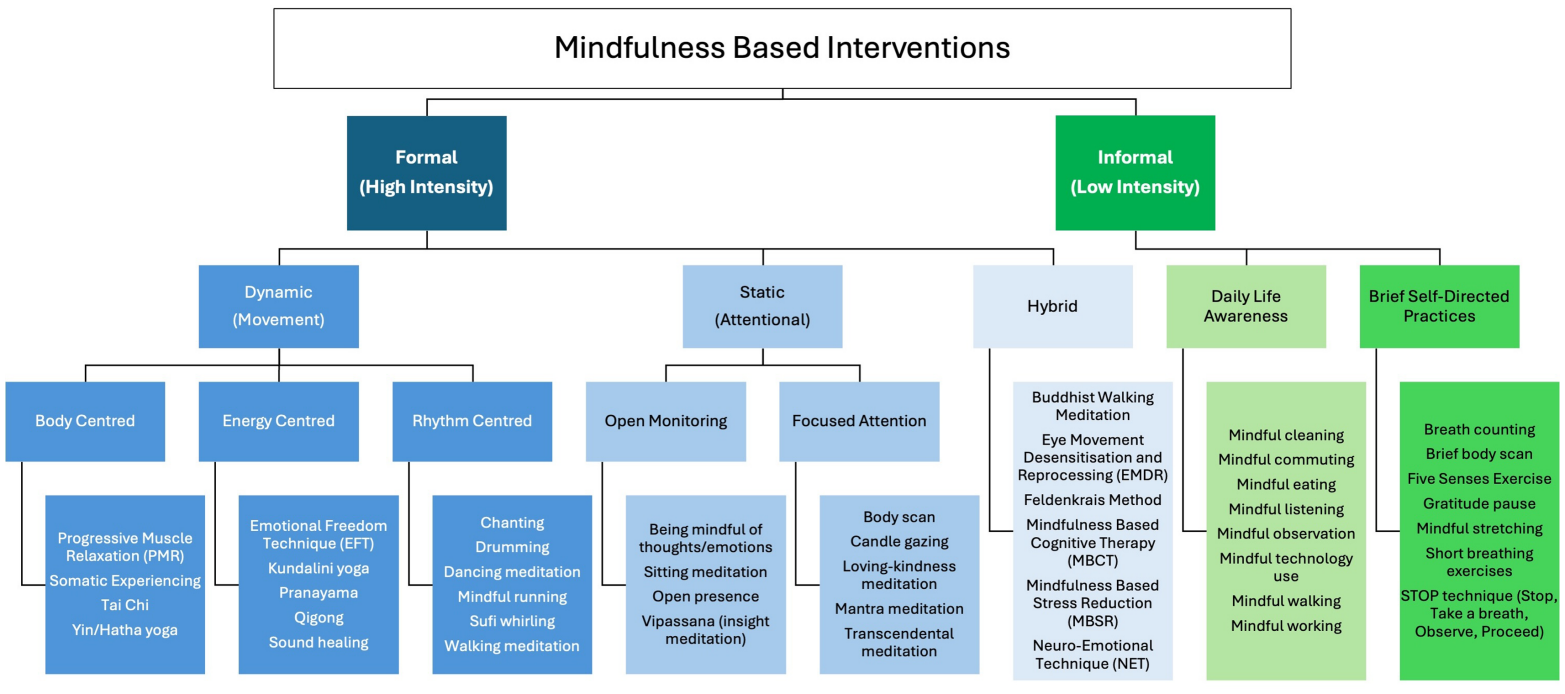
Categorisation of mindfulness based interventions (MBIs) including common examples.

Figure 2 provides a visual representation of the various categories and of MBIs, followed by some examples of each classification.

### Benefits of mindfulness-based interventions (MBIs)

Considering the pervasive health effects of chronic stress that anecdotally present in the practices of MSK practitioners, it is important to identify robust and reliable methods for counter-acting and relieving chronic stress alongside physical therapies. MBIs are practices that cultivate awareness, focus(attention), emotional regulation and acceptance of self and the present moment, with the goal of reducing chronic stress and enhancing well-being (Garland et al., 2017; Hölzel et al., 2011).

While the research assessing the effectiveness of mindfulness is continuing to evolve, and a greater number of high quality studies with larger sample sizes are needed (Zhang et al., 2021), a growing body of evidence supports the efficacy and safety of MBIs in addressing the physical and psychological effects of chronic stress (Carlson, 2012; Cardle et al., 2023; Pérez Fernández et al., 2022; Brown and Jones, 2013).

At the same time, the broader MBI literature is not without limitations. Systematic reviews consistently highlight heterogeneity in study designs, variability in intervention protocols, and modest effect sizes across conditions (Zhang et al., 2021; Ong et al., 2024). Publication bias and limited long-term follow-up data further constrain the strength of conclusions (Goldberg et al., 2022). In addition, while Table 1 summarises counter-stress effects across physiological systems, the current evidence does not permit firm conclusions about which types of MBIs (e.g., formal vs. informal, static vs. dynamic) are most efficient in producing these results (Preissner et al., 2025; Kakoschke et al., 2021). Comparative trials that directly examine format and delivery characteristics remain limited, and this represents an important area for future research.

**Table 1:** Chronic stress effects on body systems and the counter-stress effects of MBI.

Body system	Effects of chronic stress	Mechanisms of stress effects	Counter-stress effects of MBIs
Nervous system	Dysautonomia (Lucini et al., 2005), Neurotransmitter imbalances (Roth et al., 1988; Gray et al., 1989), Structural remodelling of brain areas (Mariotti, 2015; Hölzel et al., 2011; Lucassen et al., 2014; McEwen, 2007; Franklin et al., 2012; Gee et al., 2013; McEwen and Gianaros, 2011)	Chronic stress can cause dysregulation of the autonomic nervous system, leading to persistent “fight or flight” responses, fatigue, and exhaustion. It can also create neurotransmitter imbalances (e.g., dopamine, serotonin), affect brain structures like the pre-frontal cortex, and contribute to mood disorders such as depression or anxiety.	Enhanced cognitive functioning (McGreevey, 2011), structural brain changes in areas related to attention and emotional regulation (Tang et al., 2015), improved emotional regulation (Fox et al., 2014)
Endocrine system	Increased adrenaline and cortisol (Herman et al., 2016) leading to Hormonal dysregulation (Tsigos et al., 2000), Sleep disruption (Agorastos et al., 2020), Weight gain (Tsigos et al., 2000), Loss of bone density (Tsigos et al., 2000)	Prolonged stress activates the HPA axis, causing elevated cortisol levels, which may suppress immunity, accumulate visceral fat, and disrupt endocrine functions, affecting sleep, mood, digestion, reproductive health, cognitive function, and bone density.	Improved cortisol regulation (Pascoe et al., 2017), improved sleep quality (Black et al., 2015) and reduced fatigue (Zeng et al., 2019), reduced epigenetic expression of neuroendocrine dysfunction associated with type 2 diabetes (Yang et al., 2021)
Cardiovascular system	Heart disease and hypertension (Hemingway and Marmot, 1999; Gu et al., 2012), Increased risk of heart attack and stroke (Morey et al., 2015)	Stress-induced hormone release (e.g., adrenaline) can lead to heart disease, high blood pressure, and rhythm disturbances. Chronic stress also contributes to atherosclerosis, increasing the risk of heart attacks and strokes	Improved blood pressure (Thind et al., 2017; Wu et al., 2019; Gathright et al., 2019), reduced stress reactivity (Davis et al., 2015), reduced inflammation (Creswell et al., 2012; Black et al., 2013), improved lipid profile and BMI (Thind et al., 2017)
Immune SYSTEM	Inflammation (Mariotti, 2015; Black, 2002; Gu et al., 2012), Cancer (Kiecolt-Glaser et al., 2002), Autoimmunity (Stojanovich, 2010), Immunosuppression (Mariotti, 2015; Cacioppo et al., 1998)	Chronic stress overstimulates the neuroendocrine-immune axis, causing low-grade inflammation and heightened susceptibility to diseases (e.g., diabetes, cancer, auto-immune conditions) and mental health disorders. Increased HPA activation leads to immunosuppression, raising vulnerability to infections and slowing wound healing.	Reduced inflammation (Creswell et al., 2012; Black et al., 2013), enhanced immune function (Black and Slavich, 2016; Kaliman et al., 2014)
Digestive system	Inflammatory bowel conditions (IBS/IBD) (Collins, 2001; Labanski et al., 2020), Delayed gastric emptying and dysbiosis (Masere et al., 2009; Taché et al., 2001), disruption of gut microbiome (Chang et al., 2024)	Stress can cause or exacerbate gastrointestinal conditions like IBS and inflammatory bowel disease, affect digestive processes (e.g., motility, gastric emptying), and lead to dysbiosis (microbiota imbalance).	Improved biomarkers associated with IBD (González-Moret et al., 2020), increased gut microbial diversity (Wang et al., 2022)
Musculoskeletal system	Stress-related muscle tension (McFarlane, 2007), Arthritis (Buscemi et al., 2019), Pain (Finestone et al., 2008), Decreased bone density (Tsigos et al., 2000)	Chronic stress can increase muscle tension, causing pain and discomfort in areas like the back, neck, and shoulders. It can also lead to reduced bone density due to prolonged cortisol elevation, raising fracture risk.	Reduced pain and stress reactivity and better pain management (Davis et al., 2015; Riegner et al., 2024), reduced inflammation (Creswell et al., 2012; Black et al., 2013)
Metabolic system	Insulin resistance (Alam et al., 2016; Ahmed et al., 2015), Obesity (Chrousos, 2000; Streeten, 1993)	Stress can impair insulin sensitivity, contributing to Type 2 diabetes and conditions like Alzheimer’s (often called “Type 3 Diabetes”). Hormonal changes from stress can also increase cravings for high-sugar, high-fat foods, promoting weight gain.	Improved glucose and lipid metabolism (García-Campayo et al., 2018), enhanced glycaemic control (Thind et al., 2017; Dallman et al., 1993), reduced inflammation (Creswell et al., 2012; Black et al., 2013)
Reproductive system	Reduced fertility (Edwards et al., 2019), Menstrual irregularities and hormonal imbalances (Tsigos et al., 1999), Loss of Libido (Kyrou and Tsigos, 2008)	Stress affects fertility in both genders, with stress hormones disrupting menstrual cycles and reducing libido.	Reduced anxiety and depression (Spinelli et al., 2019), potential benefits in reproductive health (Ng et al., 2019), fertility (Li et al., 2016) and pregnancy (Dhillon et al., 2017; Abera et al., 2024), may reduce menopause-related stress (Liu et al., 2023)
Respiratory system	Asthma (Sandberg et al., 2000; Landeo-Gutierrez and Celedón, 2020), Hyperventilation (Suess et al., 1980), Increased Respiratory Infections (Graham et al., 1986; Drummond and Hewson-Bower, 1997)	Chronic stress can exacerbate respiratory conditions like asthma by triggering airway inflammation and hyperresponsiveness. It can also lead to hyperventilation and an increased frequency of respiratory infections due to a weakened immune response.	Reduced anxiety and stress (exacerbating factors for asthma) (Spinelli et al., 2019), reduced hyperventilation through breathwork (Balban et al., 2023), reduced respiratory infections (Zgierska et al., 2013; Barrett et al., 2012), improved asthma related clinical outcomes (Higgins et al., 2022)
Integumentary system	Hair Loss (Zhang et al., 2020), Skin disorders (e.g., Acne, Psoriasis) (Zhang et al., 2024; Chen and Lyga, 2014), Delayed wound healing (Gouin and Kiecolt-Glaser, 2012; Kiecolt-Glaser et al., 1995)	Stress affects the skin by increasing inflammatory processes and impairing the skin barrier function, leading to conditions such as acne and psoriasis. Additionally, chronic stress delays wound healing and may cause hair loss.	Reduced stress reactivity (Davis et al., 2015), better emotional regulation (Fox et al., 2014), improved inflammatory function (Black and Slavich, 2016), reduce inflammatory skin conditions (Graubard et al., 2021) such as psoriasis (Bartholomew et al., 2022) and atopic eczema (Harfenstellar, 2022)
Psychological wellbeing	Anxiety and depression (Mariotti, 2015; Won and Kim, 2016; Leonard, 2010; Kim et al., 2022), Cognitive decline (McEwen, 2007; Teixeira et al., 2015)	Chronic stress is a major risk factor for anxiety and depression due to chronic inflammation. It can also impair cognitive functions, such as memory, focus, and decision-making, due to the negative effects of stress hormones on the hypothalamus.	Reduced anxiety, depression, and distress (Kabat-Zinn et al., 1992; Hofmann et al., 2010; Spinelli et al., 2019; Ng et al., 2019), improved cognitive functioning (McGreevey, 2011), overall mental wellbeing (Gu et al., 2015), improved relationships (Carson et al., 2004), improved emotional regulation (González-Díaz et al., 2017; Fox et al., 2014), increased participation in healthy lifestyle behaviours (Rod et al., 2009)
Genome and Epigenome	Epigenetic modifications (Arzate-Mejia et al., 2024; Lutz et al., 2021; Muka et al., 2016), Altered Gene Expression (Cerniauskas et al., 2019; Musaelyan et al., 2020), Accelerated Aging (Yegorov et al., 2020; O'Donovan et al., 2012), Intergenerational health effects due to maternal gestational stress (Sandman et al., 2011; Cottrell and Seckl, 2009)	Stress can lead to epigenetic changes that alter gene expression without modifying the DNA sequence itself. These changes may accelerate aging processes and increase susceptibility to various diseases by affecting the regulation of genes involved in inflammation and immune response.	Epigenetic modifications counteracting stress effects (Gapp et al., 2016; McCreary et al., 2016), delayed biological aging (Horvath, 2013; Hannum et al., 2013; Chaix et al., 2017; Ren et al., 2012), enhanced genetic expression (Kaliman et al., 2014; Chaix et al., 2020).

Table 1 outlines the effects of chronic stress on different body systems and highlights current and emerging research of the counter-stress effects of MBIs.

### When to use caution with MBIs

**Figure 3:**
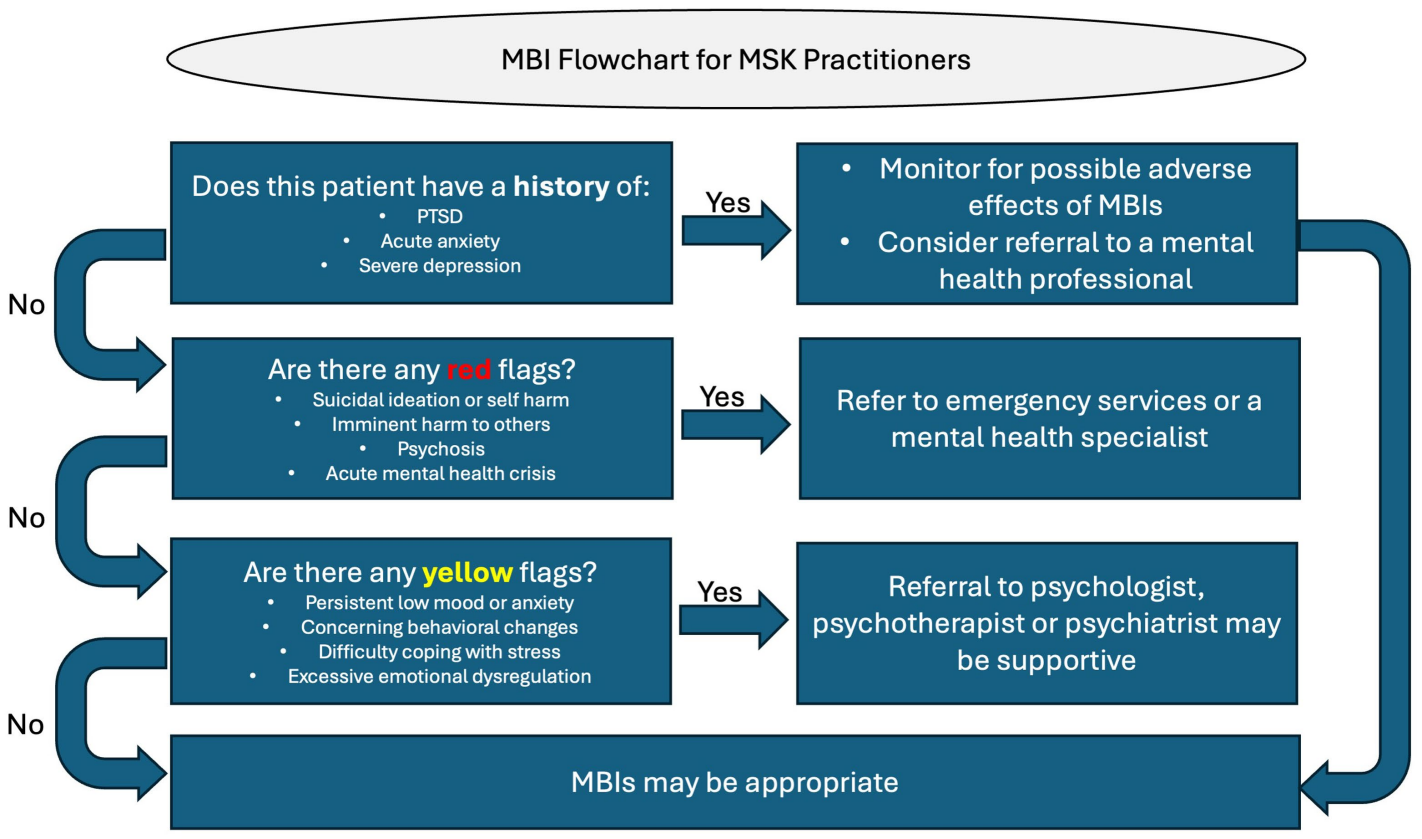
Decision flowchart for MSK practitioners when considering MBIs.

While MBIs have numerous benefits for countering the effects of chronic stress, they may not be universally suitable for everyone (Britton et al., 2021). For some individuals, particularly those with certain severe mental health conditions, MBIs can potentially trigger adverse effects. Identifying patients who exhibit “red” and “yellow” flags (Lustyk et al., 2009) is essential for making appropriate recommendations, as represented in our MSK decision flowchart (Figure 3).

Red flags are serious health indicators that require referral to a mental health specialist or emergency services, such as imminent harm to self or others, suicidal ideation, or self-harm. The MBSR standards of practice recognise this by excluding any participant with a psychological condition or suicidality (Santorelli, 2014; Dobkin et al., 2012). Research indicates that mindfulness practices may not be appropriate for people with severe psychological or psychiatric disorders, including but not limited to post-traumatic stress disorder (PTSD), acute anxiety, psychosis or severe depression (Van Dam et al., 2018; Creswell, 2017). (In contrast to older literature, more recent studies suggest that MBIs may indeed be beneficial for many sufferers of psychosis (Ellett, 2024), a reminder that this is still an evolving area of scientific enquiry).

Yellow flags include symptoms such as persistent low mood, emotional dysregulation and difficulty coping with stress. These indicators may be detected through clinical history taking or observation and, although less severe that red flags and adverse events (Binda et al., 2020), still require cautious monitoring by practitioners (Lindahl et al., 2017). Co-management with an appropriately qualified mental health professional should also be considered. Although some temporary emotional discomfort may be expected on the pathway to achieving the true benefits of mindfulness meditation (Binda et al., 2020; Lindahl et al., 2020) and should not be considered as harmful side effects (Baer and Kuyken, 2016), in a small number of cases self-reflective activities could exacerbate mental health symptoms, potentially leading to emotional numbness or distress, withdrawal, dissociation, increased anxiety, or even a resurgence of traumatic memories (Davidson and Kaszniak, 2015; Kuyken et al., 2022; Britton et al., 2021).

Therefore, before recommending MBIs it is important for MSK practitioners to implement clinical judgment and exercise appropriate caution in accordance with their scope of practice and duty of care (Farias and Wikholm, 2016). Careful patient selection and monitoring of patient responses for adverse reactions is essential to ensure that any emerging psychological complications are promptly identified and managed (Kuyken et al., 2008). To provide safe and effective care, MSK practitioners may consider establishing a referral network with qualified mental health professionals and seek to maintain open communication with these specialists when managing patients with complex psychological needs. Additionally, validated clinical tools known as Patient Reported Outcome Measures (PROMS) can assist with monitoring emotional wellbeing including the Depression, Anxiety and Stress Scale (DASS-21); (Antony et al., 1998; Lovibond and Lovibond, 1995), the Perceived Stress Scale (PSS) (Cohen et al., 1983), the Five Facet Mindfulness Questionnaire (FFMQ) (Baer et al., 2006), the Fear Avoidance Beliefs Questionnaire (FABQ) (Waddell et al., 1993), the Patient Health Questionnaire (PHQ-9) (Kroenke et al., 2001) and the Distress and Risk Assessment Method (DRAM) (Main and Waddell, 1991; Main et al., 1992). Taking a proactive approach ensures that patients receive appropriate and timely support, safeguarding both their physical and mental well-being.

### Decision-making in MSK practice

In addition to recognising red and yellow flags, MSK practitioners must also exercise decision-making that balances clinical judgment, patient preference, and scope of practice. Indicators such as stress-related symptom exacerbation, high levels of catastrophising, maladaptive coping behaviours, or a history of stress-linked pain episodes may suggest suitability for adjunctive MBIs, whereas severe psychiatric illness, active trauma symptoms, or clear patient resistance may signal the need for referral instead. Practitioner training, access to referral networks, and resource availability further shape these decisions. By applying structured screening together with these broader clinical considerations, MBIs can be integrated safely and effectively into MSK practice as adjuncts to physical therapies.

For patients with no red or yellow flags, MSK practitioners can offer guidance to patients by suggesting MBIs that might be most appropriate according to the patient’s circumstances, personality and lifestyle by considering the individual needs, conditions, and histories and applying clinical judgement (Britton, 2019). This could involve providing recommendations for mindfulness resources or practices to try, referrals to suitably qualified mental health or mindfulness practitioners, or offering adjunct mindfulness-oriented techniques alongside, or in addition to, MSK therapies.

### Neuro-emotional technique (NET) as an MBI for MSK therapies

Building on these findings, it is valuable to consider a practitioner-facilitated hybrid-MBI that exemplifies how such counter-stress effects can be integrated into musculoskeletal practice. Among the many MBIs, Neuro-Emotional Technique (NET) is highlighted here because it uniquely operationalises these principles within MSK practice through a practitioner-facilitated, structured method. Like the MBIs presented earlier in Table 1, NET has been associated with counter-stress effects across multiple systems, including neuroendocrine, immune, and musculoskeletal pathways, as highlighted below. It is therefore highlighted in this review as a representative model of how a practitioner-facilitated hybrid-MBI may operate within MSK care.

NET is a precision body–mind intervention (PBMI) (Bablis et al., 2024) that was developed by chiropractor Dr. Scott Walker in the 1980s using a 15-step methodology (Walker, 1996). Taught exclusively to registered health professionals, it is designed to identify and resolve emotional stress patterns known as Neuro-Emotional Complexes (NECs) (Walker, 1996), which are theorised to drive adverse physiological responses contributing to chronic pain and illness. NET combines elements from Traditional Chinese Medicine (TCM), Cognitive–Behavioural Therapy (CBT), and manual therapy (Foa and Jaycox, 1999), and employs the validated manual muscle test (Pollard et al., 2005; Cuthbert and Goodheart, 2007; Monti et al., 1999) as a biofeedback tool to detect stress-related responses. Recent publications suggest that NET reduces allostatic load (Bablis et al., 2024; Bablis et al., 2024) by addressing dysregulated stress response systems (HPA and PINE) (Stapelberg et al., 2015; Nold and Allers, 2020) already outlined earlier in this review. By resolving the emotional components of stress underlying chronic musculoskeletal pain and illness (Sharif et al., 2018), NET complements conventional physical therapies within a biopsychosocial model of care.

### Mindfulness mechanisms and therapeutic process of NET

While the full 15-step NET procedure has been described in Walker’s manual (Walker, 1996) and reproduced in clinical trial protocols (Karpouzis et al., 2009), a condensed overview illustrates the therapeutic flow. NET typically involves: (1) identifying a stress-related physiological response via manual muscle testing, (2) linking this response to an unresolved emotional experience (the Neuro-Emotional Complex), (3) facilitating mindful awareness and cognitive reframing of the stressor, (4) applying a brief somatic intervention (such as spinal or acupoint stimulation) during recall, and (5) re-testing to confirm resolution (Walker, 1996; Karpouzis et al., 2009). This flow illustrates how NET engages somatic awareness, mindful attention, and cognitive reappraisal – mechanisms that underpin established mindfulness interventions.

Importantly, NET’s therapeutic elements can also be understood as cultivating Kabat-Zinn’s attitudinal foundations of mindfulness (Kabat-Zinn, 2024). Meridian-based somatic cueing (derived from TCM) directs non-judgmental awareness to bodily sensations and supports letting go. Cognitive–behavioural strategies such as reappraisal and exposure foster acceptance, patience, and non-striving. Manual therapy anchoring and practitioner touch cultivate trust, beginner’s mind, and embodied presence. Together, these components operationalise mindfulness attitudes in ways that are directly relevant to MSK practice (Table 2).

**Table 2:** Integration of NET components with mindfulness attitudes and MSK clinical relevance.

NET Component	Mindfulness attitude(s)	Clinical relevance in MSK care
Somatic cueing (TCM-informed)	Non-judging, letting go, awareness	Reduces vigilance and muscle tension
CBT-style reappraisal/exposure	Acceptance, patience, non-striving	Decreases catastrophising and fear-avoidance
Manual therapy anchoring and touch	Trust, beginner’s mind, embodied presence	Improves interoception and adherence to active care

NET incorporates elements from TCM, CBT, and manual therapy. Collectively these components embody all nine attitudes of mindfulness (non-judging, awareness, letting go, acceptance, patience, non-striving, trust, beginner’s mind, gratitude/generosity), each targeting mechanisms relevant to MSK pain and stress.

During the NET process some patients appear to experience what could be described as a brief sympathetic activation or “storm,” often characterised by heightened arousal responses such as sweating, increased heart rate, or a sudden sense of insight into the origin of their stress. We hypothesise that this state reflects a momentary convergence of multiple neural systems. Specifically, the procedure may engage the subconscious or autonomic components of the nervous system (through manual muscle testing), limbic circuits involved in emotional awareness and processing, and neocortical networks implicated in contextual appraisal and meaning-making. When these elements align, the patient may experience what we have termed the Somato-Limbic Integration Point (SLIP) - a transient integrative state where bodily sensation, affective awareness, and cognitive reframing co-occur.

Although the SLIP is currently a theoretical construct, it provides a framework for describing the rapid shifts in awareness sometimes observed in clinical practice. Preliminary neuroimaging research on NET supports the plausibility of such integrative mechanisms, with Monti et al. (2017, 2018) reporting changes in activation within limbic and cortical regions following treatment. Future studies will be needed to empirically test the SLIP hypothesis and clarify its neurobiological underpinnings.

### Evidence and limitations of NET research

Research on NET has demonstrated positive outcomes across diverse conditions, including chronic low back pain (Bablis et al., 2022, 2023; Bablis and Pollard, 2016), pregnancy-related low back pain (Peterson et al., 2012), neck pain (Bablis et al., 2008), hypothyroidism (Bablis et al., 2024; Bablis and Pollard, 2004; Bablis and Pollard, 2009a), Polycystic Ovarian Syndrome (PCOS) (Bablis et al., 2006a), infertility (Bablis et al., 2006b), type 2 diabetes (Bablis et al., 2024), anxiety and depression (Jensen, 2010; Bablis and Pollard, 2009b), high cholesterol (Peterson, 1995), separation anxiety (Karpouzis et al., 2008), phobia resolution (Jensen and Ramasamy, 2009; Peterson, 1997) and ADHD (Karpouzis et al., 2009). Functional neuroimaging studies further suggest that NET influences brain physiology by reducing activation in regions associated with traumatic memories and distress (e.g., anterior cingulate gyrus, parahippocampus, insula, and brainstem) (Monti et al., 2017; Monti et al., 2018). In a randomized controlled trial of chronic low back pain, patients receiving NET in addition to standard care reported greater reductions in pain and disability (Oswestry Disability Index), lower pro-inflammatory cytokine levels (TNF-*α*, CRP, IL-1, IL-6), and improved quality of life (SF-36 domains) compared to standard care alone (Bablis et al., 2022).

To date, no published trials have reported unequivocally null or negative effects of NET on MSK outcomes. While the available evidence is encouraging, it remains preliminary, with modest sample sizes, study heterogeneity, and limited independent replication. These limitations highlight the importance of cautious interpretation and the need for further high-quality trials, including active-comparator and long-term follow-up studies, to establish NET’s role more definitively.

### Clinical relevance of NET to MSK practice

Even so, NET is particularly relevant to MSK practitioners because it addresses the psychosocial stressors that can underlie or perpetuate pain. By offering a structured method for identifying and resolving emotional stress responses in the clinical setting, NET provides a practical way to support holistic care.

Addressing the mind–body interface may help clinicians avoid missing important contributors to persistent pain and dysfunction, while integrating interventions such as NET enables MSK practitioners to address both the physical manifestations and the stress-related mechanisms that sustain them. Taken together, these findings position NET as a practitioner-facilitated, hybrid-MBI that operationalises mindfulness principles and may be feasibly integrated into MSK care.

## Conclusion

The profound interconnection between physical and mental health supports the need for a holistic approach in musculoskeletal (MSK) care. This review addressed three aims: (1) to show how chronic stress contributes to MSK conditions across biological, psychological, and social domains; (2) to synthesise evidence that mindfulness-based interventions (MBIs) can mitigate stress-related mechanisms and provide a scientific rationale for their use alongside MSK therapies; and (3) to present Neuro-Emotional Technique (NET) as an exemplar of a practitioner-facilitated hybrid-MBI, illustrating how such approaches may be applied in practice. Collectively, the evidence suggests that stress and unresolved emotional factors are not peripheral to MSK presentations but may substantially influence pain and dysfunction. Incorporating MBIs offers one pathway to address these influences, provided practitioners apply clinical judgement, implement red and yellow flag screening, and collaborate with mental health professionals where needed. While further high-quality research is required to strengthen the evidence base, integrating MBIs thoughtfully into MSK care has the potential to improve recovery, foster resilience, and support long-term health within a biopsychosocial model of care.

## Glossary

ACE: Adverse Childhood Experiences
BPS: Biopsychosocial Model of Healthcare
CBT: Cognitive Behavioural Therapy
CRP: C-Reactive Protein
DASS-21: Depression, Anxiety and Stress Scale
DRAM: Distress and Risk Assessment Method
ELS: Early Life Stress
FABQ: Fear Avoidance Beliefs Questionnaire
FFMQ: Five Facet Mindfulness Questionnaire
IL-1: Interleukin-1
IL-6: Interleukin-6
HPA: Hypothalamic–Pituitary–Adrenal
LBP: Low Back Pain
MAPs: Mindful Awareness Programs
MBI: Mindfulness-Based Intervention
MBSR: Mindfulness-Based Stress Reduction
MSK: Musculoskeletal
NEC: Neuro-Emotional Complex
NET: Neuro-Emotional Technique
ODI: Oswestry Disability Index
PBMI: Precision Body–Mind Intervention
PHQ-9: Patient Health Questionnaire
PINE network: Psycho-Immune-Neuroendocrine network
PROMs: Patient Reported Outcome Measures
PSS: Perceived Stress Scale
PTSD: Post-Traumatic Stress Disorder
RCT: Randomised Controlled Trial
SF-36: Short Form Health Survey
SLIP: Somato-Limbic Integration Point
TCM: Traditional Chinese Medicine
TNF-α: Tumour Necrosis Factor-α
